# The Usefulness of Web-Based Communication Data for Social Network Health Interventions: Agent-Based Modeling Study

**DOI:** 10.2196/44849

**Published:** 2023-11-22

**Authors:** David J Blok, Bojan Simoski, Thabo J van Woudenberg, Moniek Buijzen

**Affiliations:** 1 Erasmus School of Social and Behavioural Sciences Erasmus University Rotterdam Rotterdam Netherlands; 2 Centre for Language Studies Radboud University Nijmegen Netherlands; 3 Behavioural Science Institute Radboud University Nijmegen Netherlands

**Keywords:** agent-based modeling, peer nomination network data, physical activity, social network analysis, social network interventions, web-based communication network data

## Abstract

**Background:**

Social network interventions are an effective approach to promote physical activity. These interventions are traditionally designed using self-reported peer nomination network data to represent social connections. However, there is unexplored potential in communication data exchanged through web-based messaging apps or social platforms, given the availability of these data, the developments in artificial intelligence to analyze these data, and the shift of personal communication to the web sphere. The implications of using web-based versus offline social networks on the effectiveness of social network interventions remain largely unexplored.

**Objective:**

This study aims to investigate the differences in the impact of social network interventions on physical activity levels (PALs) between networks derived from web-based communication and peer nomination data.

**Methods:**

We used the data on sociometric questionnaires, messages from a web-based communication app, and PAL (number of steps per day) of 408 participants in 21 school classes. We applied social network analysis to identify influential peers and agent-based modeling to simulate the diffusion of PAL and explore the impact of social network interventions on PAL among adolescents in school classes. Influential peers (n=63) were selected based on centrality measures (ie, in-degree, closeness, and betweenness) to spread the intervention. They received health education, which increased their PAL by 17%. In sensitivity analyses, we tested the impact of a 5%, 10%, and 20% increase in PAL among influential peers.

**Results:**

There was a 24%-27% overlap in selected influential peers between the 2 network representations. In general, the simulations showed that interventions could increase PAL by 5.0%-5.8% within 2 months. However, the predicted median impact on PAL was slightly higher in networks based on web-based communication data than peer nomination data for in-degree (5.7%, IQR 5.5%-6.1% vs 5.5%, IQR 5.2%-5.8%; *P*=.002), betweenness (5.6%, IQR 5.4%-5.9% vs 5.0%, IQR 4.7%-5.3%; *P*<.001), and closeness centrality (5.8%, IQR 5.6%-6.1% vs 5.3%, IQR 5.0%-5.6%; *P*<.001). A large variation in impact was observed between school classes (range 1.5%-17.5%). Lowering the effectiveness of health education from 17% to 5% would reduce the overall impact of the social network intervention by 3-fold in both networks.

**Conclusions:**

Our findings showed that network interventions based on web-based communication data could increase PAL. Web-based communication data may therefore be a valuable addition to peer nomination data for future social network intervention design. Artificial intelligence methods, including agent-based modeling, can help to design these network interventions and provide insights into the role of network characteristics in their effectiveness.

## Introduction

Social networks can influence health behaviors significantly, as people and their health behaviors are known to be connected [1-3]. Youth are particularly susceptible to influences from role models in their social networks (eg, family and peers), who can encourage or discourage health behaviors. For example, studies have shown that peers and peer groups shape consumption behavior and physical activity among youth [4-7]. Developments in social network theory have provided opportunities to exploit social networks as a tool to promote positive behaviors, such as physical activity [8]. Network interventions are rooted in the diffusion of innovation theory [9], which presumes that ideas and behaviors spread through a social network. A common approach in designing social network interventions is to identify influential peers (ie, role models) based on network properties, such as centrality measures [8]. The selected individuals can then be educated and trained to promote healthy behaviors among their peers [9].

The selection of influential peers is traditionally based on social network representations derived from self-reported peer nomination data [10]. Participants are asked to name and rank peers based on questions such as “who are your close friends?” [4]. Although such networks can provide a relatively realistic representation of real-life relationships, they have some known limitations. First, self-reporting of peers could result in underreporting of relationships with undesired peers (social desirability bias) or overestimating relationships with whom they interacted recently (recall bias) [11,12]. Second, survey questions may be interpreted differently and therefore result in an incomplete or nonrepresentative network. Third, nonresponse is a serious concern, as questions to derive a nominated network can be considered tedious [13]. Nonresponse can result in gaps in the network that may affect the understanding of the network, and, as a result, the intervention design (eg, misinterpreting centrality measures). Finally, as nominated networks only focus on the most important relationships, weak ties may be overlooked. Weak ties can play an important role in diffusing information or behaviors in a network [14,15].

Communication data from web-based social platforms or other forms of web-based media communication could provide a useful addition for measuring social relationships [16-18]. Connections and interactions on web-based social platforms can be used to create a representation of web-based social networks. A connection on web-based social platforms is only meaningful when interactions between peers have taken place [18]. The number of interactions between peers can more accurately measure the quality of a relationship and opportunities to exchange information and can therefore be a good proxy for assigning the strength of ties (ie, weighted ties) in a social network. Artificial intelligence (AI) methods, such as natural language processing (NLP) techniques, could be used to filter meaningful conversations and calculate weights. Web-based social networks have also become an attractive target for promoting healthy behaviors [19,20]. Web-based relationships can provide supportive interactions to encourage healthy behaviors and can increase an individual’s physical activity through social comparison [19,21,22].

Studies have shown that web-based and offline social networks capture (to a large extent) different connections between adolescents [23,24]. This may suggest that web-based and peer nominated social networks measure different types of relationships, which would likely affect the design of social network interventions (ie, selection of influential peers). As a result, the effectiveness of these interventions in promoting healthy behaviors may be different as well. There is little knowledge about the consequences of social network interventions design and effectiveness when based on web-based communication data as compared to peer nomination data.

Therefore, this study aims to investigate the differences in impact of social network interventions between networks derived from web-based communication and peer nomination data. We examined the effectiveness of social network interventions to promote physical activity, where interventions are designed based on web-based communication data, and compared it to peer nomination data. We apply social network analysis (SNA) to a longitudinal data set collected among school classes in the Netherlands [25]. This data set contains information about participants’ physical activity levels (PALs), the socioeconomic status of their family, peer-nominated social contacts, and web-based text messages exchanged between classmates. To assess the effectiveness of social network interventions, we apply agent-based modeling, a powerful tool combined with AI to address complex problems, as an alternative to real-world trials. An agent-based model (ABM) can conduct experiments in simulated environments to assess the causal effect of interventions on health behaviors [26]. ABMs are increasingly being used to compare the impact of various social network intervention strategies (ie, selection of influential peers) [27-30], and to investigate the potential (long-term) impact of network interventions on health behaviors, such as physical activity behavior [31,32]. In this study, we present an ABM to simulate the diffusion of physical activity among youth in social networks and use it to compare the effect of social network interventions on PALs in networks derived from web-based communication and peer nomination data.

## Methods

### Participants

We used the data from the MyMovez research project investigating youth’s social networks and health behaviors [25]. The participants were children and adolescents, between 8 and 15 years of age, from 21 Dutch primary and secondary schools. They received a smartphone with a research app to fill out questionnaires, including sociometric questionnaires about the relationships with and impressions of other classmates. The app also included a web-based social platform for communication between peers. Furthermore, participants received a wrist-worn accelerometer to measure PALs.

The data were collected during a 3-year period in 7 data waves: February 2016 (W1), April 2016 (W2), June 2016 (W3), February 2017 (W4), February 2018 (W5), April 2018 (W6), and June 2018 (W7). Each data wave lasted for 7 days. The web-based social platform was introduced in W4 of the project, but this wave had a high attrition rate and participants leaving schools. In W5, new classrooms were added, and the web-based social platform was used by 617 out of 736 (84%) participants. Therefore, we used W5 to infer social networks from both web-based communication and peer nomination data and to measure PAL and family affluence scores. W1 was only used to assign the initial PAL of the participants in the model.

We included participants who both filled out the sociometric questionnaires and used the web-based social platform in W5 (614 participants in 44 classes). Only participants from classes with more than 60% participation and at least 15 participants were included to ensure a representative sample within each class [33]. In total, our sample included 408 adolescents in 21 school classes (19 primary and 2 secondary). The mean age was 10.6 (SD 1.0) years, and 220 (54%) participants were female (Multimedia Appendix 1).

### Measures

#### Social Networks

##### Web-Based Communication Data

Web-based social networks in school classes were created using communication data from the web-based social platform in the research app in W5. Participants could post messages on the message board of the classroom or send private messages to classmates on the web-based social platform. To create a network of personal relationships, we only used private (one-to-one) messages between participants within a class (*c*). Every message *m* sent by a participant *i_c_* (ego) to a classmate who received the message *j_c_* (alter) was considered a connection, resulting in a directed network. The weight of a connection between 2 peers was based on the total number of messages agent *i_c_* (ego) receives from peer *j_c_* (alter) and on the maximum number of peer-to-peer messages in a class *max* (*m_c_*). The total weight of a connection in the communication network between an agent *i_c_* (ego) and *j_c_* (alter) in class *c* is given by:



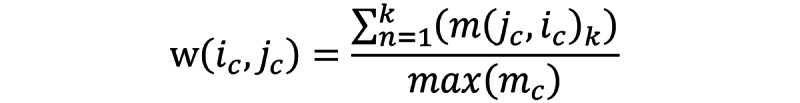



##### Peer Nomination Data

Social networks in school classes were created based on peer nomination data derived from sociometric questions. In W5, 4 general sociometric questions were asked, including (1) who they hang out or have contact with [5]; (2) who they go to for advice; (3) who they consider as leaders; and (4) who they want to be like [34]. Participants received these 4 questions *N*(*q*) through the research smartphone at a random time during the day. For each question (*q*), participants were required to nominate at least one peer, with no maximum on the nominated peers, and self-nominations were not possible. We only considered nominations from within a class (*c*). Question 1 was used to generate a directed network with edges from the participant *i_c_* (ego) to a nominated peer *j_c_* (alter), because it mostly resembles web-based exchanged messages between peers. The weight of a connection, which reflects the amount of influence that an ego has on an alter, was based on all 4 nomination questions. The weight of a connection in the nominated network between agents *i_c_* (ego) and *j_c_* (alter) is derived as follows:



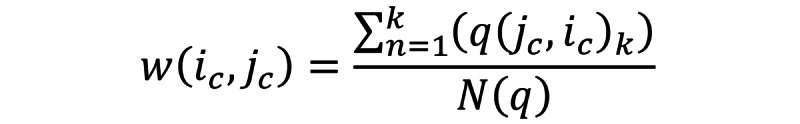



#### PAL Measure

PALs were measured using the accelerometer (Fitbit Flex), which participants wore for 7 consecutive days in each wave. We excluded days 1 and 7 because the data on these days were not full days of measurement by default. Also, days with less than 24 hours of data collection and days with less than 1000 steps were excluded because these were incomplete data (eg, nonwear time). The PAL was calculated as the mean of steps per day for at least three days of valid data in a wave. For participants with less than 3 days of valid data, the number of steps per day was imputed using single multilevel (predictive mean matching) imputation. Missing data were imputed based on other physical activity data of the same participant, day of the week, measurement period, sex, and age [35]. The number of steps per day of an individual was divided by 10,000. The mean PAL was 0.92 (minimum 0.12 and maximum 1.87) in W5 (Multimedia Appendix 1).

#### Family Affluence Scale

The Family Affluence Scale (FAS) is a self-reported measure of the socioeconomic status of participants’ families [36,37]. This measure was included in our ABM to account for the impact of socioeconomic circumstances on PALs. Participants were asked the following four questions: (1) does your family own a car, van, or truck? (2) Do you have your own bedroom for yourself? (3) During the past 12 months, how many times did you go on a holiday with your family? (4) how many computers does your family own? The scores of all possible answers were summed, with higher scores representing a better socioeconomic position (range 0-12). The mean score was 9.1 (minimum 2 and maximum 12; Multimedia Appendix 1).

### Procedure

#### SNA Procedure

SNA was performed on both social networks to investigate network structures and select influential peers based on centrality measures. SNA was conducted using the package *NetworkX* 2.8 in Python 3 (Python Software Foundation).

Both social networks were compared based on number of connections, average connection weight, network density, and a similarity score. Network density indicates the level of cohesion in a network and is measured as the ratio between the number of connections in a network and the number of all possible connections. To measure the similarity between social networks derived from web-based communication and peer nomination data, we used the Jaccard similarity index. It divides the number of connections in a class shared between both social networks by the total number of connections (shared and unshared). The Jaccard similarity index will be 0 if none of the affiliating node pairs co-occurs in both social networks and 1 if both networks are identical in connections.

#### Selection of Influential Peers

Social network interventions engage influential peers to disseminate behavior change. Influential peers are typically selected based on centrality measures (ie, network position), because it is assumed that being central in a network makes an individual more influential [8]. There are various ways to measure centrality. In this study, social network interventions were based on 3 common centrality measures for selecting influential individuals: in-degree, betweenness, and closeness centrality [38].

In-degree centrality refers to the number of connections directed toward an individual. This measure is related to the number of peer nominations or web-based messages an individual receives. Individuals with higher in-degree centrality can be seen as important holders of information and are perceived as the most popular in school settings [38].

Betweenness centrality is a measure of centrality based on how often an individual is part of the shortest paths between all nodes in a network. It is often used to find individuals that link different subgroups together, so-called “bridge” individuals. This means that an individual with high betweenness centrality controls the flow of information between other peers in the network. If the betweenness central agents are not selected as influential peers, entire subgroups might be restrained from the intervention [38].

Closeness centrality provides a measure of how close an individual is to all other peers in a network. Individuals with the highest closeness centrality values have, on average, the shortest path to all other peers in a network. This means that the intervention will reach the entire network in the lowest number of steps.

In this study, the top 15% (63/408) of individuals with the highest centrality score were selected as influential peers in each class. The number of influential peers selected per class ranged between 2 and 4 (depending on the class size) in both network representations. In case multiple individuals had a similar centrality score, these individuals were ranked on highest PAL. If there was still a tie, the influential peer was randomly selected.

#### Agent-Based Modeling

##### Model Description

An ABM was developed to describe the diffusion dynamics of physical activity through a social network. Our model was built upon previously published ABMs by van Woudenberg et al [30] and de Mello Araujo et al [39], which were based on the model framework by Beheshti et al [29] and Giabbanelli et al [40]. The model was programmed in Python. Model source code and scripts are available on GitHub [41].

In the model, each agent is characterized by a PAL and socioeconomic environment score (ie, the FAS score). An agent’s PAL may change over time as a result of two key factors: (1) the influence through the social network and (2) influence of socioeconomic environment. At each time step (ie, day), an agent determines whether to change his or her PAL. To change PAL, the impact of social influence and the socioeconomic environment should exceed a threshold (*T_PAL_*), which is the minimum amount of influence an agent needs to receive to change behavior. If the condition is true, the agent increases or decreases his or her PAL by a factor *I_PAL_* (Multimedia Appendix 2 for full description).

##### Model Calibration

A grid search was used to calibrate two model parameters (*T_PAL_* and *I_PAL_*). The model was calibrated using the modeled social network based on web-based communication and peer nomination data separately. Both models were run until predictions had reached a steady state. Initial PALs of each agent were randomly sampled from the distribution of PALs by sex in W1 (Multimedia Appendix 3). The simulated PALs in the steady state were compared to the empirical data per class in year 1 (W2-W3), year 2 (W4), and year 3 (W5-W7). The goodness-of-fit was measured by the sum of squared errors. The objective function was to minimize the sum of squared errors. We selected 100 best-fitting model parameter combinations for both social network representations separately. In this study, all simulations were run using these parameter combinations (ie, 100 runs) to account for uncertainty in the parameter estimates. Multimedia Appendix 2 provides a full description of the calibration procedure.

##### Simulation of Social Network Interventions

The calibrated model was used to simulate the impact of hypothetical social network interventions on PAL in social networks based on web-based communication and peer nomination data separately. The social network intervention featured health education among the top 15% (63/408) most influential peers, which would motivate them to increase their PAL (eg, through intensive personal counseling). In the model, we assumed that the health education intervention would increase the PAL of influential peers by 17%. This artificial increase was based on previous modeling studies [29,30]. In addition, we assessed the impact of an increase of 5%, 10%, and 20%. We tested three strategies to select influential peers for the social network intervention: (1) in-degree, (2) betweenness, and (3) closeness centrality measure (see Selection of Influential Peers section).

We simulated the social network intervention strategies for an additional 200 days from the calibrated model outcomes. Each social network intervention strategy was simulated 100 times (ie, for each calibrated parameter combination). For each run, we calculated the average PAL over all agents in the model. We presented the median from the 100 simulation runs. The variation in impact between runs (ie, uncertainty interval) was presented in a box plot. The primary outcome measure is the relative impact of each network intervention on the mean PAL per class per day. The relative impact represents the difference in PAL at the start (day 0) and the end (day 200) and was expressed as a percentage of change.

### Ethical Considerations

Informed consent was obtained from 1 of the parents of the participants in the MyMovez project. Study procedures were approved by the Ethics Committee of the Radboud University (ECSW2014–100614-222).

## Results

### SNA Procedure

Table 1 and Multimedia Appendix 3 present the overall characteristics of web-based communication and peer nomination network data per school class. In the web-based communication network data, a total of 25,739 messages were exchanged among all participants. The number of messages exchanged ranged from 143 to 5301 between classes. Multimedia Appendix 4 provides information about the distribution of the exchanged messages. The number of connections per individual varied significantly among classes (mean 8, SD 6). In the peer nomination network data, there were a total of 3063 peer nominations among the 21 classes (mean 146, SD 59). There were on average 8 (SD 5; in and outgoing) connections per individual, with a variation among the classes ranging from 4 to 12 connections per individual on average.

**Table 1:** Characteristics of web-based communication and peer nomination data.

Characteristic			Value
Participants, n			408
**Web-based communication data**			
	Web-based messages, n	25,739^a^	
	Number of connections per participant, mean (SD)	8 (6)	
	Average connection weight, mean (SD)	0.10 (0.12)	
	Network density, mean (SD)	0.38 (0.18)^b^	
**Peer nomination data**			
	Peer nominations, n	3063^c^	
	Number of connections per participant, mean (SD)	8 (5)	
	Average connection weight, mean (SD)	0.43 (0.14)	
	Network density, mean (SD)	0.40 (0.09)^b^	
Network similarity, mean (SD)			0.69 (0.09)

^a^The mean number of web-based messages per school class is 1225 (SD 1309).

^b^No significant difference between web-based and peer nominated social networks (*P*=.47; Mann-Whitney *U* test).

^c^The mean number of peer nominations per school class is 146 (SD 59).

The overall average network density was similar in web-based social networks (0.38) and peer nominated social networks (0.40). The average connection weight was 0.10 (SD 0.12) in web-based social networks compared to 0.43 (SD 0.14) in peer nominated social networks. The overall similarity between networks based on web-based communication compared to peer nomination data was 0.69 (ie, 5253/7700 connections appeared in both networks). Overall, networks based on peer nomination data had significantly higher closeness (0.56 vs 0.54) centrality than networks based on web-based communication data (Multimedia Appendix 5). The overall in-degree (0.40 vs 0.39) and betweenness (0.03 vs 0.04) centrality was similar in both network representations. Participant-level SNA is available in Multimedia Appendix 6, providing comparative insights from an ego-level perspective.

### Selection of Influential Peers

**Figure 1:**
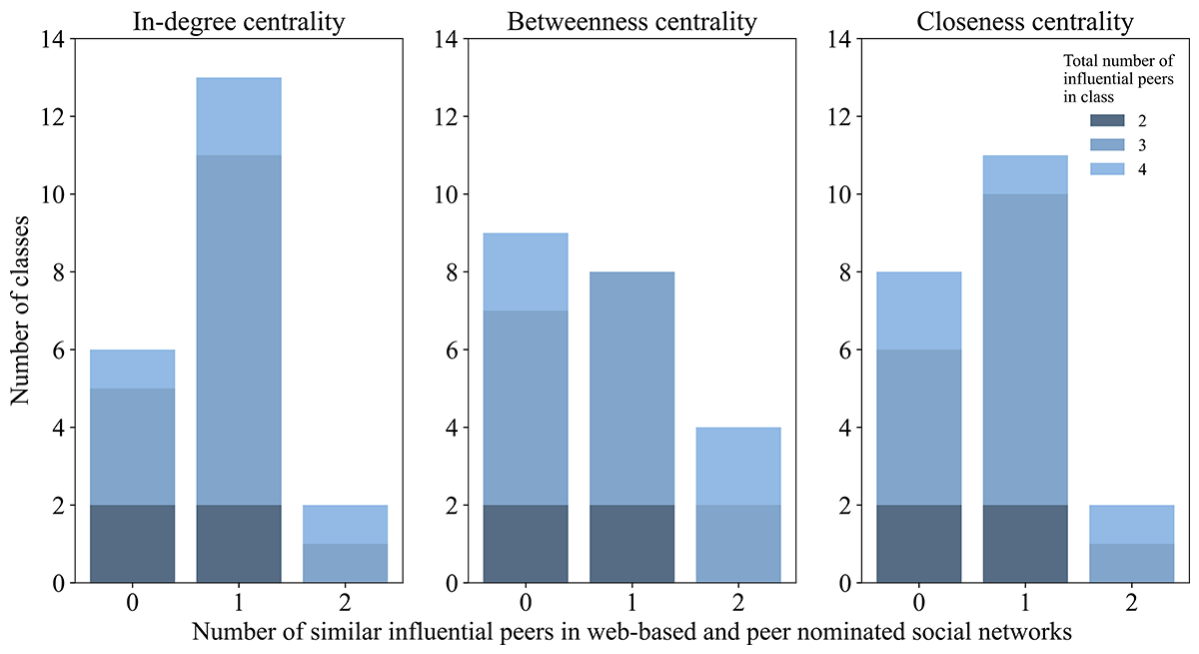
Frequency distribution of the number of similar influential peers in web-based and peer nominated social networks per class.

The majority of the selected influential peers were different in networks based on web-based communication and peer nomination data. On average, 17/63 (27%; in-degree), 16/63 (25%; betweenness), and 15/63 (24%; closeness) of the selected influential peers were the same in both networks. In more than 6 classes, the selected influential peers were completely different (Figure 1). In web-based social networks, in-degree and closeness centrality had an 86% (54/63), in-degree and betweenness had 63% (40/63), and betweenness and closeness had 67% (42/63; Multimedia Appendix 7) overlap in influential peers. In peer nominated social networks, this was 81% (51/63; in-degree vs closeness), 44% (28/63; in-degree vs betweenness), and 43% (27/63; betweenness vs closeness).

### Agent-Based Modeling

**Figure 2:**
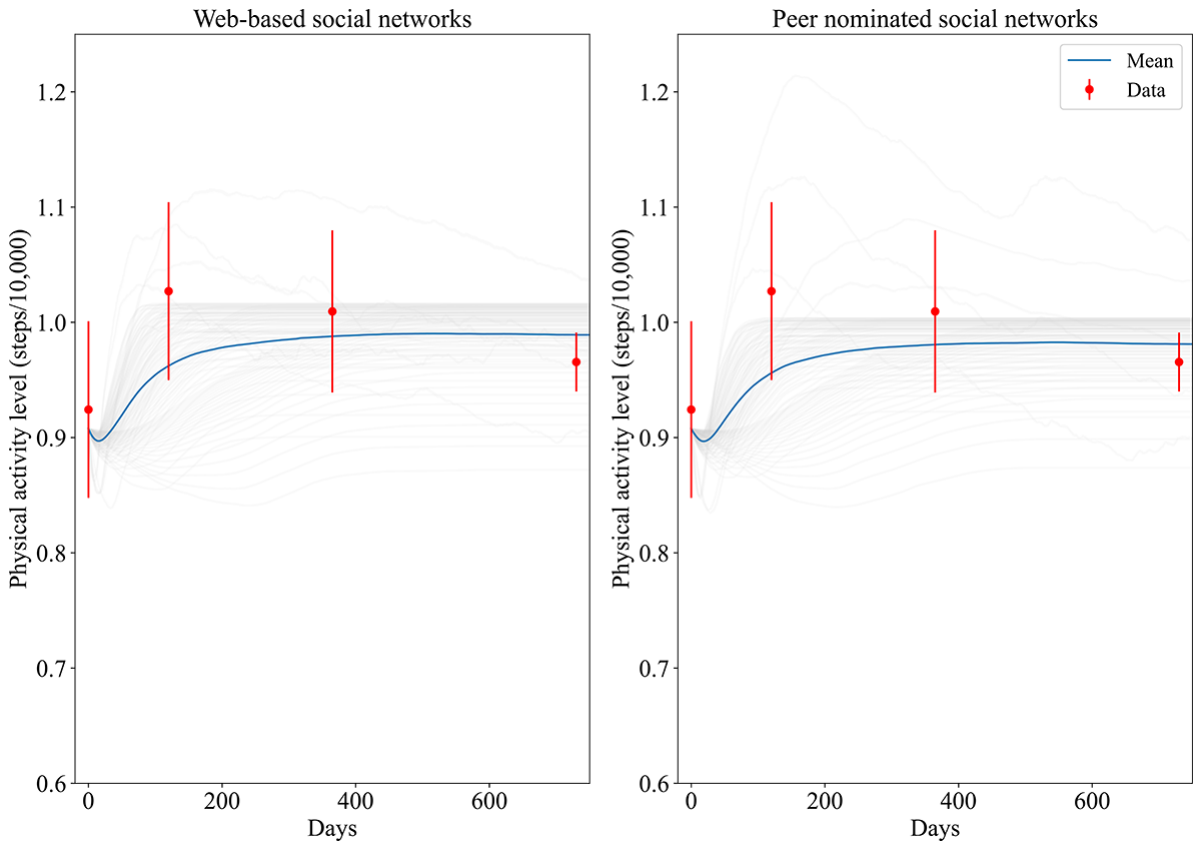
Results of calibrating the model to the observed physical activity level.

Figure 2 shows the predicted PAL from the model (100 simulation runs and its mean) using web-based and peer nominated social networks with the observed data from all waves. The mean predictions of PAL (blue line) were within the CIs of the empirical data for all data points, indicating a good fit. The 100 simulation runs (light gray lines) indicate the variation due to parameter uncertainty, which were in the same order for both network types.

**Figure 3:**
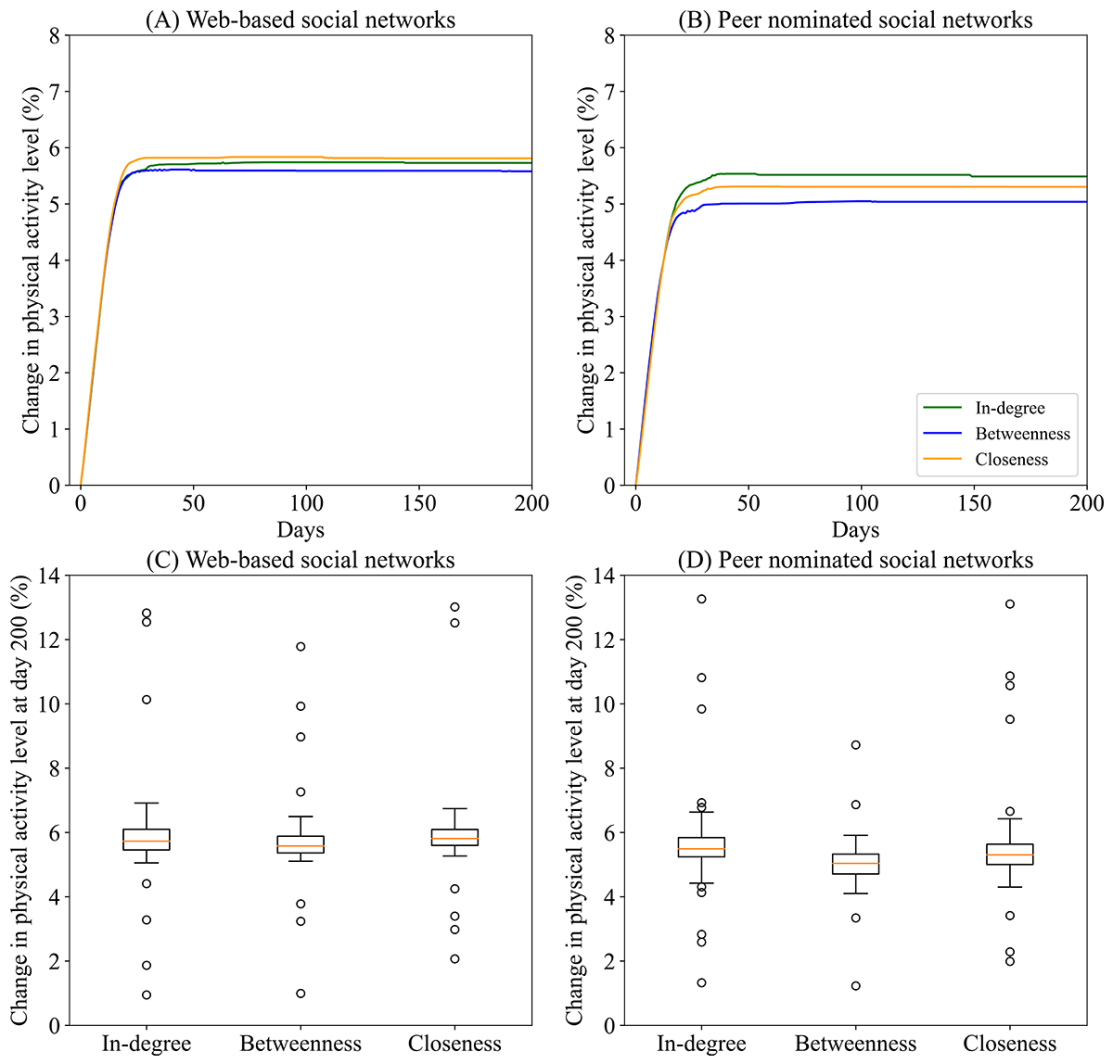
Modeled effect of social network interventions on the average physical activity level over a period of 200 days.

Figure 3 illustrates the predicted effect of network intervention strategies on the mean change in PAL among all participants in both social networks. All 3 strategies for network interventions increased the average PAL in both data network types. The median impact on PAL was higher in networks based on web-based communication than peer nomination data for in-degree (5.7%, IQR 5.5%-6.1% vs 5.5%, IQR 5.2%-5.8%; *P*=.002; Mann-Whitney *U* test), betweenness (5.6%, IQR 5.4%-5.9% vs 5.0%, IQR 4.7%-5.3%; *P*<.001), and closeness centrality (5.8%, IQR 5.6%-6.1% vs 5.3%, IQR 5.0%-5.6%; *P*<.001). In social networks based on web-based communication data, the increase in PALs corresponds with an average increase of 581, 569, and 594 steps per day when influential peers were selected based on in-degree, betweenness, and closeness centrality, respectively. In peer nominated social networks, the increase in PALs corresponds with an average increase of 561, 512, and 537 steps per day for in-degree, betweenness, and closeness centrality, respectively. The increase in PALs varied by selection strategy, with closeness showing the largest impact, followed by in-degree and betweenness using web-based social networks (Figures 3A and 3C). For peer nominated social networks, in-degree centrality showed the largest impact, followed by closeness and betweenness (Figures 3B and 3D). Multimedia Appendix 8 provides more information about the variation in impact between school classes.

**Figure 4:**
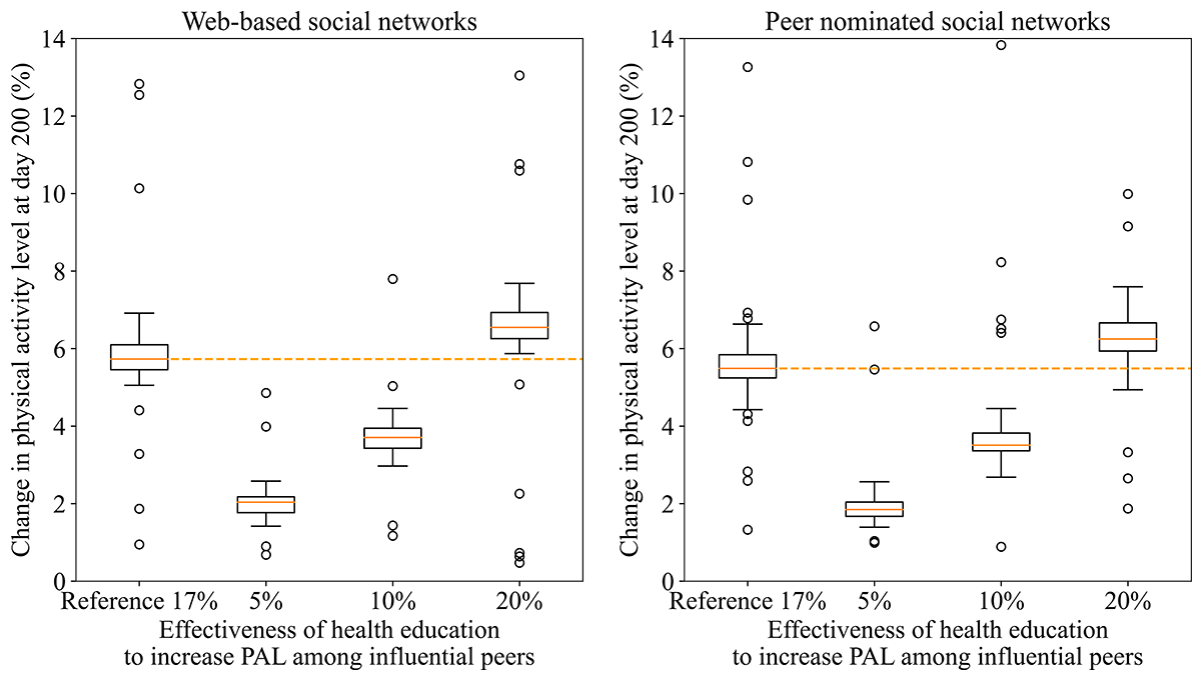
The impact of social network interventions on physical activity levels with varying effectiveness of health education to influential peers. PAL: physical activity level.

Varying the effectiveness of health education to increase PAL among influential peers (selected based on centrality measures) changed the overall impact of the social network intervention (Figure 4 and Multimedia Appendix 9). Assuming an effectiveness of 5% or 10% would yield an average population-level impact that is approximately 67% or 34% lower, respectively, than the reference (17%). Increasing the effectiveness to 20% would increase the PAL by 12%-16% compared to the reference.

## Discussion

### Overview

This study investigated the differences in impact between using web-based communication data and peer nomination data for designing social network interventions. In both network representations, interventions could increase PALs substantially (5%-5.8%) within 2 months. Generally, the predicted impact of the interventions was slightly higher in networks based on web-based communication than peer nomination data. The selection strategy for influential peers only slightly affected the impact of the social network interventions in both types of networks. However, varying the increase in PAL among influential peers (ie, the effectiveness of health education) significantly changed the impact, with lower effectiveness resulting in a lower impact.

The differences in impact using web-based communication and peer nomination network data could be explained by the differences in the constructed network. Social networks derived from web-based communication and peer nomination data showed 69% similarity, suggesting that almost one-third of the connections are different. Also, web-based social networks had significantly lower connection weights but similar density compared to the peer nominated networks. Nevertheless, the impact was slightly higher in web-based social networks. This is primarily due to substantial differences in the selection of influential peers between the 2 data representations (approximately 25% overlap). Influential peers that were selected in web-based social networks had a significantly higher number of connections than in peer nominated social networks. In 2 school classes, influential peers in web-based social networks were connected to all classmates and in 8 classes connected to more than 80% (range 19/23-20/20) of all classmates, while in peer nominated social networks, the latter occurred in only 1 school class. This suggests that being fully or very connected may compensate for the lack of strength of connection in the effectiveness of social network interventions.

As selection strategies for influential peers are usually based on network positions, choosing web-based or offline data for representing social networks is crucial. It is known that web-based and offline connections differ substantially, as peers on the internet are not necessarily the same as the closest peers offline [23,24]. This study also found substantial differences in connections in both networks, although we attempted to align both networks by selecting the nomination question (“who they hang out or have contact with”) that mostly resembles web-based exchanged messages between peers. This suggests that the frequency of web-based communication does not necessarily equal the number of peers one physically hangs out with. This should be considered when selecting web-based versus offline connections to design network interventions aimed at increasing physical activity.

Our findings showed that the choice of a particular selection strategy for influential peers (ie, in-degree, betweenness, or closeness) only slightly affected the impact on PAL in both networks. The differences were at most 0.2% in web-based social networks or 0.5% in peer nominated social networks. The relatively small variation in impact was the result of the large overlap in selected influential peers between selection strategies (ie, in-degree, betweenness, and closeness) in both networks. A plausible explanation for this large overlap could be the small social network sizes (on average, 8 connections per class in both networks). This may suggest that for relatively small network sizes, any selection strategy (ie, in-degree, betweenness, or closeness) could be used to achieve a similar positive impact.

The impact of social network interventions was very sensitive to the assumed effectiveness of health education (ie, increase in PAL among influential peers). Assuming lower effectiveness would reduce the overall impact of the social network intervention by 3-fold in both networks. This underlines the importance of developing effective methods to train and educate influential peers for social network interventions to be impactful.

In this study, networks based on web-based communication data had significantly lower weights than peer nomination data. This is inherent to the method of calculating weights and the limitations of the data sets. In peer nomination networks, the weight was based on 4 nomination questions, resulting in a weight of either 0, 0.25, 0.5, 0.75, or 1. In web-based social networks, the number of messages exchanged between individuals was used as a weight, resulting in more granular weights (approximating a continuous scale from 0 to 1). This resulted in a larger variation in the weight of the connection (right-skewed distribution). A continuous scale might provide a more realistic reflection of the importance of connection. However, although weights in peer nomination data were restricted, these weights might better reflect influential potential as the peer nomination questions were influence-oriented.

This is the first agent-based modeling study to directly compare the effect of social network interventions on PAL using web-based communication versus peer nomination data. A strength of this work is that our ABM was based on an existing model of social network diffusion of PAL that has been previously (and independently) tested [29,30,39]. Also, we calibrated the model using actual data. These data were unique as they included information on participants’ peer-nominated friendships, web-based interactions, and PALs, allowing a direct comparison between the implications of using web-based communication versus peer nomination data. Moreover, this study used a published framework for agent-based modeling studies (in the field of public health) to assess the quality of the model [42]. Our ABM complies with the principles related to data, parameters, sensitivity analysis, validation, and documentation. All parameter values used were reported, including those derived from data and distributions based on data. Calibration values for the model were also provided, ensuring transparency and accuracy in representing the underlying dynamics of the system. Sensitivity analyses were applied to the effectiveness of health education (ie, increase of PAL among influential peers) and network density. Model validation is conducted by comparing the simulation run outcomes with reference data sets, that is, the empirical data. Finally, we have fully documented the experiment for reproducibility through open data access, open-source code, and code documentation.

Nevertheless, the presented ABM could be further refined by including, for example, individual personality traits (eg, age effects), seasonality, and other relevant environmental factors (eg, neighborhood characteristics). Moreover, the temporality of the networks should be included in the design since social connections change over time, while the current model assumes static social networks. This could provide better-informed suggestions for designing interventions.

The data in this study were derived from a web-based social platform of a research app that participants could freely use, but they were not instructed or forced to use the app’s web-based platform. Therefore, while our web-based communication data may not reflect all web-based communication, it is likely that participants may also have been communicating through other social media apps with classmates during the study. As a result, connection weights might be underestimated since they were derived based on the number of limited web-based interactions. Future studies could ask the target audience to donate their social media data [43].

Another limitation is that we considered every text message exchanged between participants in the web-based communication data to be equally meaningful. However, it is conceivable that in youth’s web-based communication, this is not always the case. For example, it is very unlikely that exchanging greetings (eg, “hi”) or ending a conversation (eg, “bye”) can be used to persuade or influence someone to change his or her behavior. Similarly, more messages sent within 1 conversation cannot be directly interpreted as a more persuasive conversation. Therefore, future work should look more closely at the content of web-based interactions. For example, one straightforward improvement would be weighting the messages based on a word count instead of a message count. Another option would be to weigh connections based on how meaningful the interactions are. NLP techniques could be used to filter meaningful conversations related to a certain topic or behavior. For example, NLP techniques can analyze text messages and assign weights to network edges based on the level of interaction expressed. Lower weights could be assigned to surface-level interactions, such as simple greetings, while higher weights can represent more complex interactions, such as detailed discussions where peers react to each other’s ideas.

Clearly, web-based communication data are complex by nature and therefore allow numerous ways for more sophisticated network modeling. The data offer many opportunities for future work on representing real-life social networks and designing network interventions. On the contrary, peer nomination data describe a snapshot of close relationships, which can be considered a limitation in terms of representing real-life social dynamics. One potentially promising approach is to combine data coming from web-based communication and peer nomination sources to design more realistic multimodal network representations.

Another way to enrich the proposed network representations is by using sampling techniques to correct the social network data or applying mixed approaches. For example, snowball sampling procedures, a type of respondent-driven sampling (RDS), can be applied to sample hidden (or hard-to-reach) populations [44]. One can consider influencers as a type of hidden population within a network. RDS exploits the social network structure to reach the target population through the participants’ own peers [45]. However, instead of a static peer nomination network, with snowball sampling, the network grows as participants refer other peers. Moreover, mixed methods can also be applied. For instance, a web-based social network strategy combined with RDS peer referral has been shown to recruit a more representative sample for recruitment of a very specific group of young transwomen compared to using only RDS [46]. We imagine that in the case of the identification of influential peers, a similar mixed methods strategy combining web-based social media sources with well-established sampling techniques can lead to some promising outcomes.

### Conclusions

Altogether, the findings of this study indicate that network interventions designed based on web-based communication network data can be promising. This analysis showed that there are noticeable structural differences between the web-based communication and peer nomination network data, which affect the selection of influential peers and the outcomes of the network interventions. Generally, interventions could increase PAL substantially within 2 months in both network representations. Web-based communication data may therefore be a valuable addition and alterative to peer nomination data for future social network intervention design to overcome the disadvantages of peer-nomination-only procedures. With advances in technology, people are shifting much of their real-life communication to web-based social media. This offers an unprecedented opportunity for new ways of interpreting social connections and peer influence. AI methods, including agent-based modeling, can help to better understand these social phenomena and serve as a useful tool for designing network health interventions.

## Appendix

Multimedia Appendix 1 Demographic characteristics of the participants in wave 5 by school class.

Multimedia Appendix 2 Model description and calibration.

Multimedia Appendix 3 Characteristics of web-based communication and peer nomination data by school class.

Multimedia Appendix 4 Frequency distribution of messages in web-based communication data.

Multimedia Appendix 5 Centrality measures of generated social networks using web-based communication and peer nomination data by school class.

Multimedia Appendix 6 Distribution of participants with same connections in web-based and peer nominated social networks.

Multimedia Appendix 7 Number of similar influential peers between in-degree, betweenness and closeness.

Multimedia Appendix 8 Variation of the impact of social network interventions on physical activity levels between school classes.

Multimedia Appendix 9 The impact of social network interventions on physical activity levels with varying effectiveness of health education to influential peers.

## Abbreviations

ABM: agent-based model
AI: artificial intelligence
FAS: Family Affluence Scale
NLP: natural language processing
PAL: physical activity level
RDS: respondent-driven sampling
SNA: social network analysis

## Data Availability

The data used in this study are stored at the Data Archiving and Networked Services [47]. Model source code and scripts are available on GitHub [41].
