# Supplementary material for: The Usefulness of Web-Based Communication Data for Social Network Health Interventions: Agent-Based Modeling Study
Source: JMIR Pediatr Parent. 2023 Nov 22;6:e44849. doi: 10.2196/44849 (PMC10701651; doi:10.2196/44849)
Supplement: Multimedia Appendix 1 [file pediatrics_v6i1e44849_app1.pdf]

Multimedia Appendix 1

Table. Demographic characteristics of the participants in wave 5 by school class.

Class ID	Participants n	Age (years) mean (SD)	Sex (female) %	Physical activity level[a] mean (SD)	Family affluence score mean (SD)
1	18	11.6 (0.5)	55.6	0.87 (0.29)	8.5 (2.2)
2	19	11.5 (0.8)	52.6	0.79 (0.21)	9.1 (1.6)
3	19	11.5 (0.5)	36.8	0.88 (0.34)	8.6 (2.4)
4	17	11.3 (0.5)	64.7	0.99 (0.30)	8.8 (2.1)
5	16	11.6 (0.6)	56.3	0.98 (0.25)	9.0 (1.6)
6	22	12.2 (0.4)	54.5	0.79 (0.32)	9.8 (1.0)
7	16	12.4 (0.6)	62.5	1.02 (0.29)	10.1 (2.0)
8	17	10.3 (0.5)	35.3	1.04 (0.26)	9.2 (1.5)
9	15	10.6 (0.6)	46.7	0.93 (0.31)	9.2 (1.8)
10	20	10.5 (0.5)	65.0	0.81 (0.18)	9.7 (1.6)
11	24	9.6 (0.6)	37.5	1.01 (0.38)	8.7 (2.5)
12	24	10.5 (0.7)	50.0	0.92 (0.40)	8.9 (2.2)
13	21	9.6 (0.7)	76.2	0.88 (0.33)	9.7 (1.3)
14	17	9.7 (0.5)	70.6	0.80 (0.36)	9.2 (1.5)
15	16	10.4 (0.5)	68.8	0.95 (0.31)	9.1 (2.4)
16	21	9.4 (0.5)	57.1	0.99 (0.32)	9.8 (1.5)
17	19	10.4 (0.5)	47.4	0.96 (0.27)	9.8 (1.7)
18	19	10.5 (0.6)	52.6	0.93 (0.38)	8.6 (1.9)
19	24	9.4 (0.5)	29.2	0.93 (0.38)	7.3 (2.5)
20	19	10.8 (0.6)	68.4	0.86 (0.21)	8.1 (2.1)
21	25	10.3 (0.5)	60.0	1.02 (0.28)	9.6 (1.9)
Total	408	10.6 (1.0)	54.2	0.92 (0.31)	9.1 (2.0)

[a] Number of steps per 10,000
