# Supplementary material for: The Usefulness of Web-Based Communication Data for Social Network Health Interventions: Agent-Based Modeling Study
Source: JMIR Pediatr Parent. 2023 Nov 22;6:e44849. doi: 10.2196/44849 (PMC10701651; doi:10.2196/44849)
Supplement: Multimedia Appendix 2 [file pediatrics_v6i1e44849_app2.pdf]

Multimedia Appendix 2

Model Description

An agent-based model (ABM) was developed to describe the diffusion dynamics of physical activity through a social network. Our ABM was built upon previously published ABMs by [30, 39], which were based on the model framework by [29, 40]. The model was programmed in Python. Model source code and scripts are available on GitHub (See Data Availability).

In the model, we assume that an agent's physical activity is influenced by peers' behavior (social network influence) and affected by socioeconomic environment of the agent. Each agent is characterized by a PAL and socioeconomic environment score (i.e., the FAS score).

An agent's PAL may change over time as a result of two key factors: 1) the influence through the social network and 2) influence of socioeconomic environment. The influence of peers on an agent's PAL is modeled as follows:

$$inf_{PAL_i(t)} = \frac{\sum_j \left(PAL_j(t-1) - PAL_i(t-1)\right) \cdot w(i,j)}{\sum_j w(i,j)}$$

A positive inf_{PA_i} implies that peers have a positive impact on agent i's PAL, while a negative value means that they have negative impact. The influence level inf_{PA_i} is determined by past PALs of all peers $PAL_j(t-1)$ and his/her own level $PAL_i(t-1)$ and weighted by the strength of the connection. The influence level could further be affected by socioeconomic environment (env). We used the FAS as an indicator of env. In the model, we scaled the FAS per participant as follows: $FAS = |(score_i/6) - 2|$. This resulted in a scale from 0 to 2 (mean = 0.49, SD = 0.33). A score between 0 and 1 represents a supportive socioeconomic environment, while a score between 1 and 2 a low socioeconomic environment. The socioeconomic environment (env) affects the inf_{PAL_i} as follows:

$$inf_{PAL_i(t),\,env} = \begin{cases} env \cdot inf_{PAL_i(t)}, & if\ inf_{PAL_i(t)} < 0 \\ \frac{1}{env} \cdot inf_{PAL_i(t)}, & if\ inf_{PAL_i(t)} \geq 0 \end{cases}$$

At each time step (i.e., day) an agent determines whether to change his/her PAL. In the model, we assume that the impact of social influence and the socioeconomic environment should exceed a threshold (T_{PAL}) to change PAL: $|inf_{PAL_i(t), env}| \geq T_{PAL}$. This threshold can be considered as the amount of influence an agent needs to receive to change behaviors. If the condition is true, the agent increases or decreases his/her physical activity level by a factor I_{PAL} :

$$PAL_i(t) = \begin{cases} (1 + I_{PAL}) \cdot PAL_i(t-1), & if\ inf_{PAL_i(t),env} \geq 0 \\ (1 - I_{PAL}) \cdot PAL_i(t-1), & if\ inf_{PAL_i(t),env} < 0 \end{cases}$$

If $|inf_{PAL_i(t), env}| < T_{PAL}$, the agent's PAL remains unchanged: $PAL_i(t) = PAL_i(t-1)$. T_{PAL} and I_{PAL} were calibrated such that simulated PALs match empirical data.

Model Calibration

Two model parameters were calibrated (T_{PAL} and I_{PAL}) using a grid search. The model was calibrated using the modeled social network based on web-based communication and peer nomination data separately. Both models were run until predictions had reached a steady state. Initial PALs of each agent were randomly sampled from the distribution of PALs by sex of W1 (Figure 1). The simulated PALs in the steady state were compared to the empirical data per class in Year 1 (W2-3), Year 2 (W4) and Year 3 (W5-W7). The goodness-of-fit was measured by the sum of squared errors (SSE). The objective function was to minimize the SSE.

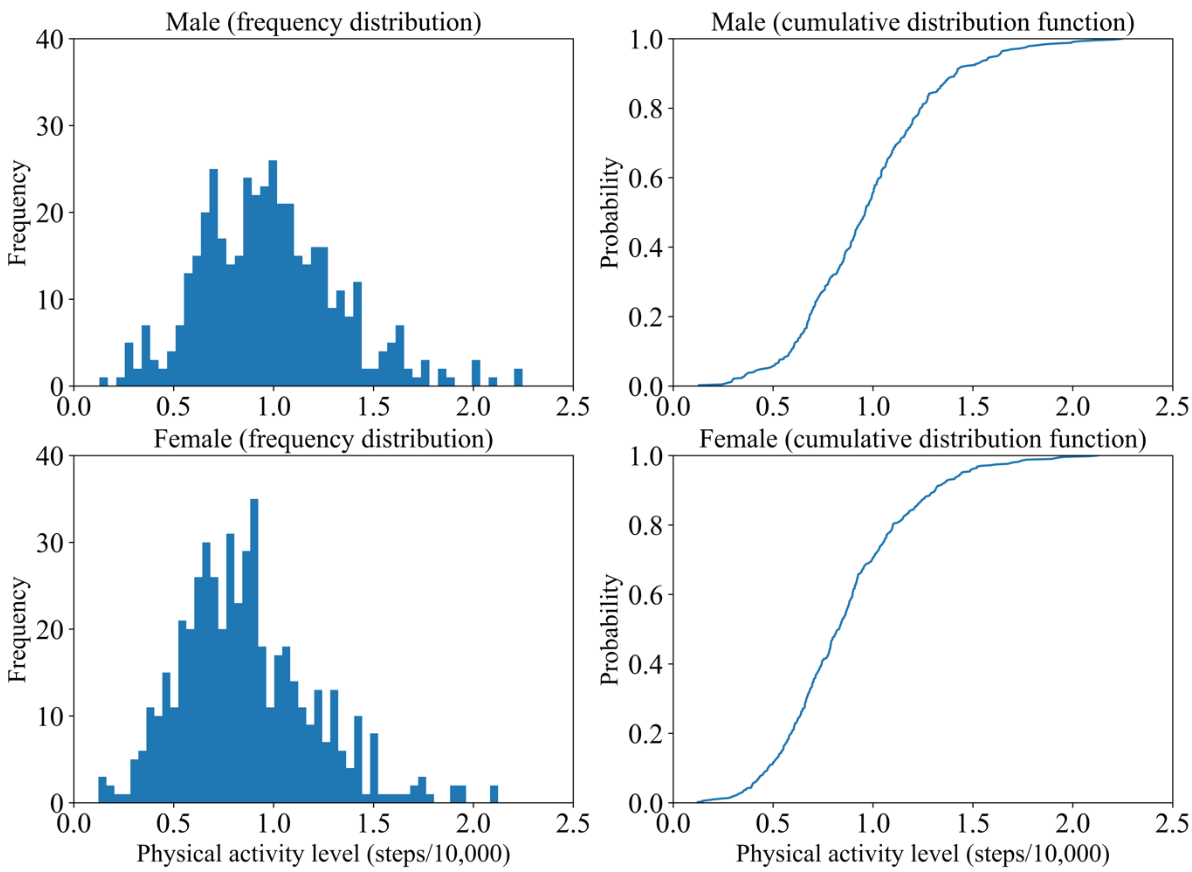

Figure 1. Distribution of physical activity levels by sex in wave 1. *Physical activity levels represent the number of steps/10,000. Initial physical activity level of each agent in the model was sampled from this distribution for male and female separately. 83% of the participants of wave 5 (study population) were not included in wave 1.*

First, a preliminary grid search was performed to narrow down the parameter space. We varied each parameter ranging from 0.001 to 1.00 with increments of 0.05 to find a subspace of parameter combinations that would provide a good fit, i.e., smallest SSE (Figure 2A and 2B), and that would result in at least 80% (n>236) of participants changing behaviors over time (Figure 2C and 2D). This resulted in a subspace of: $0 < T_{PA} \leq 0.1$ and $0 < I_{PA} \leq 0.05$ for both models (ie, web-based and peer nominated social network model). Afterwards, we conducted a grid search using the identified subspace, varying T_{PA} and I_{PA} with steps of 0.0025 (Figure 3A and 3B). We selected 100 best fitting model parameter combinations for both social network representations separately (Figure 3C and 3D).

In this study, all simulations were run using these parameter combinations (i.e., 100 runs) to account for uncertainty in the parameter estimates.

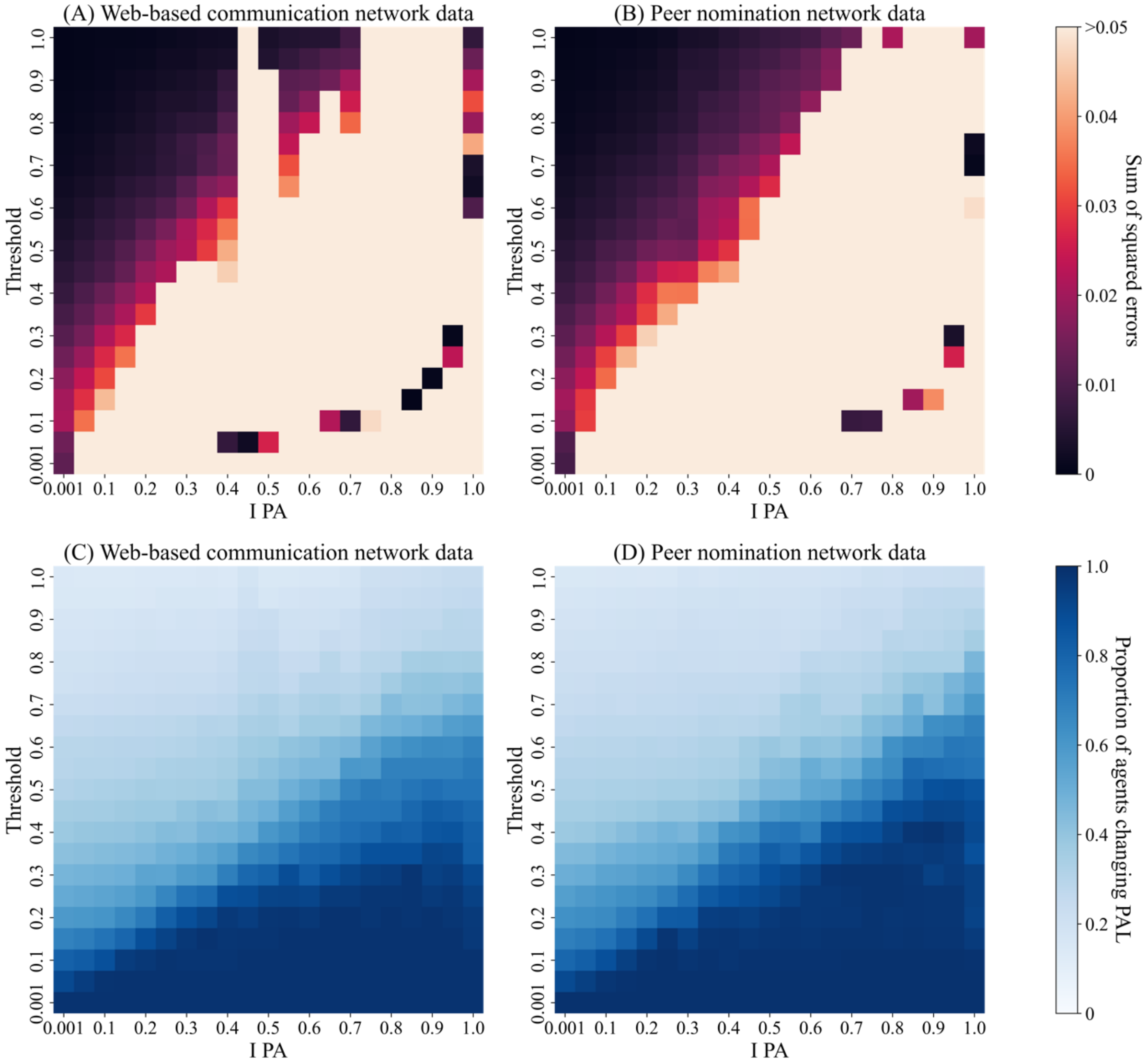

Figure 2. Results of preliminary grid search. *An initial grid search was performed to narrow down the parameter space. The threshold (T_{PA}) and influence (I_{PA}) parameter were varied ranging from 0,001 to 1 with steps of 0.05. Panels A and B show the goodness of fit, i.e., sum of squared errors (SSE), for simulations based on web-based communication and peer nomination data, respectively. Colors indicate the SSE score ranging from 0 (black) to >0.05 (lighter color). Panels C and D show the proportion of agents that changed physical activity levels (i.e., number of steps/10,000) during the simulation. Darker blue colors indicate a higher proportion of agents changing physical activity levels.*

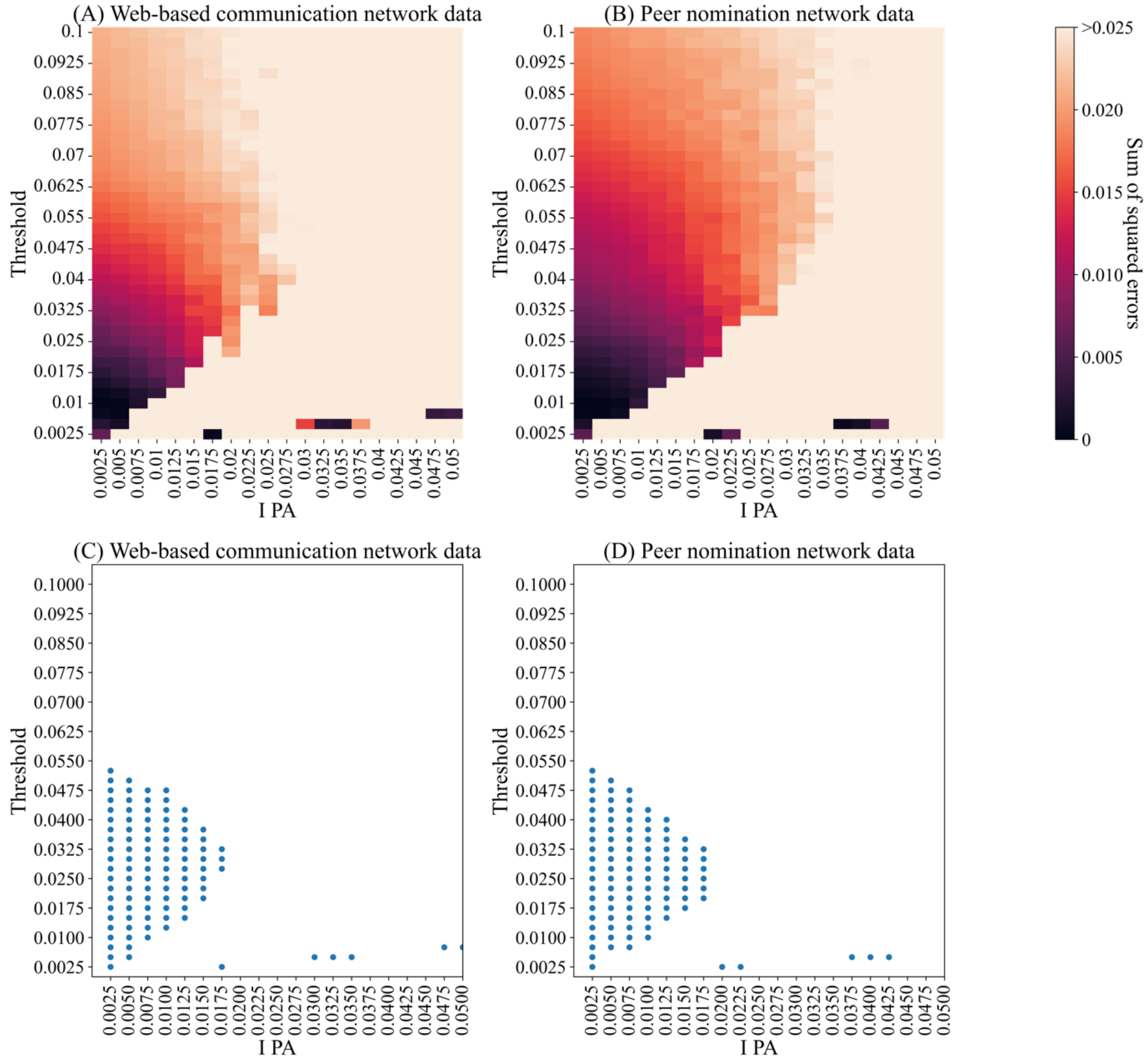

Figure 3. Results of final grid search. *A grid search was performed varying the threshold (T_{PA}) and influence (I_{PA}) parameters with steps of 0.0025, resulting in 800 parameter combinations. Panels A and B show the goodness of fit, i.e., sum of squared errors (SSE), for simulations based on web-based communication and peer nomination data, respectively. Colors indicate the SSE score ranging from 0 (black) to >0.025 (lighter color). Panels C and D indicate the 100 best-fitting parameter combinations, i.e., lowest SSE. These parameter combinations were used in the final simulations.*
