# Supplementary material for: The Usefulness of Web-Based Communication Data for Social Network Health Interventions: Agent-Based Modeling Study
Source: JMIR Pediatr Parent. 2023 Nov 22;6:e44849. doi: 10.2196/44849 (PMC10701651; doi:10.2196/44849)
Supplement: Multimedia Appendix 3 [file pediatrics_v6i1e44849_app3.pdf]

Multimedia Appendix 3

Table. Characteristics of web-based communication and peer nomination data by school class

Class ID	Partici pants	Web-based communication data				Peer nomination data				Network similarity
		Online messages	Number of connections per participant	Average connection weight	Network density	Peer nominations	Number of connections per participant	Average connection weight	Network density	
	n	n	mean (SD)	mean (SD)		n	mean (SD)	mean (SD)		
1	18	221	3 (2)	0.14 (0.10)	0.14	81	5 (3)	0.34 (0.12)	0.26	0.79
2	19	143	5 (6)	0.31 (0.20)	0.21	136	7 (5)	0.46 (0.14)	0.4	0.64
3	19	569	5 (3)	0.06 (0.05)	0.27	131	7 (4)	0.46 (0.23)	0.38	0.71
4	17	356	5 (4)	0.07 (0.08)	0.28	154	9 (4)	0.48 (0.13)	0.57	0.60
5	16	561	5 (5)	0.06 (0.07)	0.3	117	7 (5)	0.39 (0.12)	0.49	0.52
6	22	259	3 (2)	0.24 (0.20)	0.14	105	5 (2)	0.5 (0.13)	0.23	0.85
7	16	168	3 (2)	0.18 (0.18)	0.14	86	6 (3)	0.39 (0.14)	0.36	0.76
8	17	448	5 (4)	0.10 (0.10)	0.33	102	6 (4)	0.47 (0.2)	0.38	0.73
9	15	263	4 (3)	0.21 (0.20)	0.3	64	4 (2)	0.46 (0.12)	0.3	0.81
10	20	1013	8 (5)	0.07 (0.08)	0.43	135	7 (4)	0.45 (0.13)	0.36	0.70
11	24	2411	12 (7)	0.06 (0.05)	0.51	259	11 (6)	0.42 (0.14)	0.47	0.63
12	24	1145	11 (8)	0.07 (0.06)	0.46	291	12 (7)	0.47 (0.12)	0.53	0.67
13	21	2168	10 (5)	0.05 (0.05)	0.48	119	6 (4)	0.48 (0.21)	0.28	0.66
14	17	1107	8 (4)	0.05 (0.04)	0.47	120	7 (5)	0.43 (0.14)	0.44	0.67
15	16	386	4 (3)	0.11 (0.08)	0.29	109	7 (4)	0.35 (0.09)	0.45	0.77
16	21	5301	16 (3)	0.05 (0.03)	0.81	172	8 (3)	0.44 (0.1)	0.41	0.56
17	19	2363	13 (5)	0.05 (0.04)	0.71	169	9 (4)	0.47 (0.17)	0.49	0.64
18	19	512	7 (4)	0.08 (0.06)	0.35	132	7 (5)	0.41 (0.09)	0.39	0.69
19	24	2508	14 (7)	0.06 (0.07)	0.61	172	7 (5)	0.42 (0.14)	0.31	0.53
20	19	731	8 (6)	0.16 (0.08)	0.46	152	8 (5)	0.44 (0.1)	0.44	0.70
21	25	3106	10 (7)	0.04 (0.05)	0.37	257	10 (6)	0.38 (0.09)	0.43	0.78
