# Supplementary material for: The Usefulness of Web-Based Communication Data for Social Network Health Interventions: Agent-Based Modeling Study
Source: JMIR Pediatr Parent. 2023 Nov 22;6:e44849. doi: 10.2196/44849 (PMC10701651; doi:10.2196/44849)
Supplement: Multimedia Appendix 4 [file pediatrics_v6i1e44849_app4.pdf]

Multimedia Appendix 4

Figure. Frequency distribution of messages in web-based communication data.

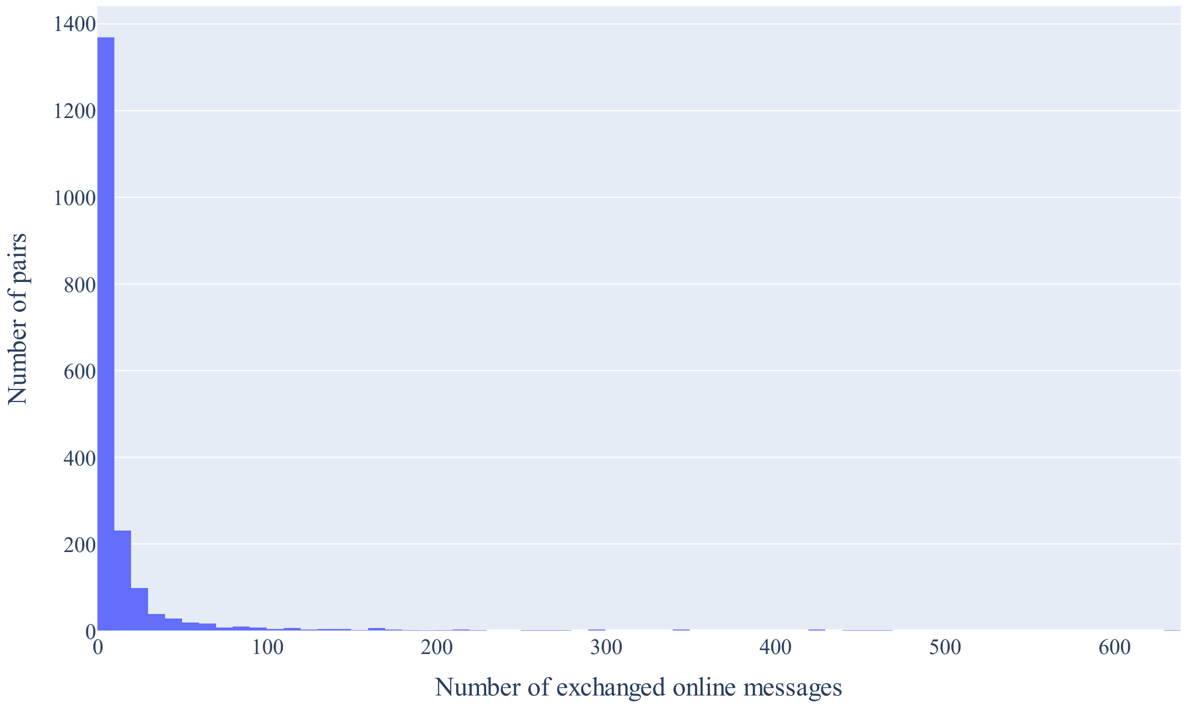

Note. The number of messages sent by a pair in the web-based communication app. Bin size is 10 messages.
