# Supplementary material for: The Usefulness of Web-Based Communication Data for Social Network Health Interventions: Agent-Based Modeling Study
Source: JMIR Pediatr Parent. 2023 Nov 22;6:e44849. doi: 10.2196/44849 (PMC10701651; doi:10.2196/44849)
Supplement: Multimedia Appendix 5 [file pediatrics_v6i1e44849_app5.pdf]

Multimedia Appendix 5

Table. Centrality measures of generated social networks using web-based communication and peer nomination data by school class.

		Web-based social network			Peer nominated social network		
Class ID	Participants n	In-degree mean (SD)	Closeness mean (SD)	Betweenness mean (SD)	In-degree mean (SD)	Closeness mean (SD)	Betweenness mean (SD)
1	18	0.14 (0.08)***	0.25 (0.12)**	0.07 (0.07)	0.26 (0.11)	0.38 (0.09)	0.10 (0.12)
2	19	0.21 (0.10)***	0.42 (0.06)***	0.07 (0.11)	0.40 (0.11)	0.57 (0.07)	0.05 (0.07)
3	19	0.27 (0.12)***	0.51 (0.07)	0.06 (0.06)	0.38 (0.15)	0.53 (0.15)	0.04 (0.04)
4	17	0.28 (0.13)***	0.50 (0.06)***	0.07 (0.13)	0.57 (0.17)	0.70 (0.09)	0.03 (0.04)
5	16	0.30 (0.14)**	0.53 (0.07)**	0.06 (0.10)	0.49 (0.13)	0.62 (0.07)	0.05 (0.05)
6	22	0.14 (0.07)***	0.32 (0.09)	0.09 (0.11)	0.23 (0.08)	0.32 (0.10)	0.04 (0.09)
7	16	0.14 (0.11)**	0.23 (0.13)*	0.03 (0.06)	0.36 (0.21)	0.39 (0.21)	0.01 (0.01)
8	17	0.33 (0.12)	0.55 (0.06)*	0.05 (0.12)*	0.38 (0.20)	0.40 (0.19)	0.01 (0.01)
9	15	0.30 (0.13)	0.50 (0.06)**	0.08 (0.15)	0.30 (0.14)	0.36 (0.13)	0.11 (0.11)
10	20	0.43 (0.09)*	0.60 (0.05)*	0.04 (0.05)	0.36 (0.10)	0.48 (0.16)	0.03 (0.05)
11	24	0.51 (0.16)	0.67 (0.08)	0.02 (0.03)	0.47 (0.10)	0.64 (0.04)	0.03 (0.03)
12	24	0.46 (0.11)	0.64 (0.06)*	0.03 (0.03)	0.53 (0.09)	0.68 (0.05)	0.02 (0.02)
13	21	0.48 (0.12)***	0.64 (0.06)***	0.03 (0.04)	0.28 (0.11)	0.43 (0.06)	0.04 (0.04)
14	17	0.47 (0.13)	0.66 (0.06)	0.04 (0.04)	0.44 (0.17)	0.62 (0.09)	0.04 (0.07)
15	16	0.29 (0.17)**	0.44 (0.16)**	0.09 (0.10)	0.45 (0.13)	0.56 (0.05)	0.02 (0.03)
16	21	0.81 (0.12)***	0.85 (0.08)***	0.01 (0.01)***	0.41 (0.11)	0.59 (0.08)	0.04 (0.03)
17	19	0.71 (0.10)***	0.78 (0.06)***	0.02 (0.01)	0.49 (0.08)	0.61 (0.07)	0.03 (0.04)
18	19	0.35 (0.17)	0.59 (0.09)	0.04 (0.07)	0.39 (0.11)	0.57 (0.05)	0.04 (0.07)
19	24	0.61 (0.11)***	0.70 (0.06)***	0.02 (0.02)	0.31 (0.12)	0.48 (0.06)	0.04 (0.04)
20	19	0.46 (0.11)	0.65 (0.05)	0.03 (0.04)	0.44 (0.13)	0.64 (0.06)	0.03 (0.05)
21	25	0.37 (0.16)**	0.60 (0.08)*	0.03 (0.05)	0.43 (0.08)	0.61 (0.04)	0.03 (0.03)
Total	408	0.39 (0.22)	0.56 (0.17)*	0.04 (0.08)	0.40 (0.15)	0.54 (0.15)	0.04 (0.06)

* *P*< .05; ** *P*< .01; *** *P*<.001; Difference between web-based and peer nominated social network (Mann Whitney U Test)
