# Supplementary material for: The Usefulness of Web-Based Communication Data for Social Network Health Interventions: Agent-Based Modeling Study
Source: JMIR Pediatr Parent. 2023 Nov 22;6:e44849. doi: 10.2196/44849 (PMC10701651; doi:10.2196/44849)
Supplement: Multimedia Appendix 6 [file pediatrics_v6i1e44849_app6.pdf]

Multimedia Appendix 6

Figure. Distribution of participants with same connections in web-based and peer nominated social networks.

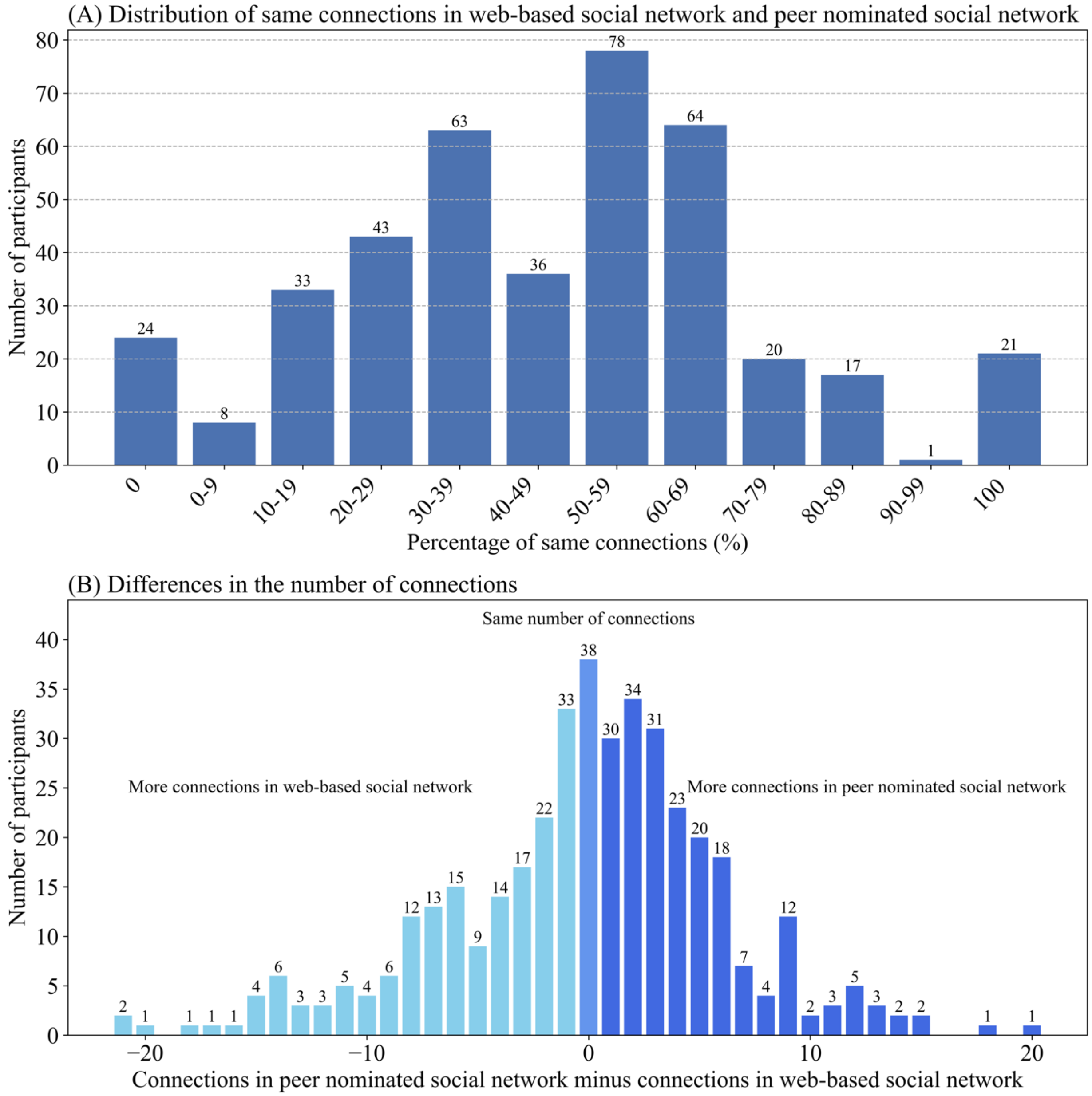

Note. Panel A shows the frequency of having the same connections in web-based and peer nominated social networks. The percentage of same connections is calculated as the number of same connections divided by the total number of connections. Panel B visualizes the difference in the number of connections in the peer

nominated and web-based social network per participant. There were 38 participants that had the same number of connections in both network representations, 198 participants had more connections in the peer nominated social network, and 172 participants had more connections in the web-based social network.
