# Supplementary material for: The Usefulness of Web-Based Communication Data for Social Network Health Interventions: Agent-Based Modeling Study
Source: JMIR Pediatr Parent. 2023 Nov 22;6:e44849. doi: 10.2196/44849 (PMC10701651; doi:10.2196/44849)
Supplement: Multimedia Appendix 7 [file pediatrics_v6i1e44849_app7.pdf]

Multimedia Appendix 7

Figure. Number of similar influential peers between in-degree, betweenness and closeness.

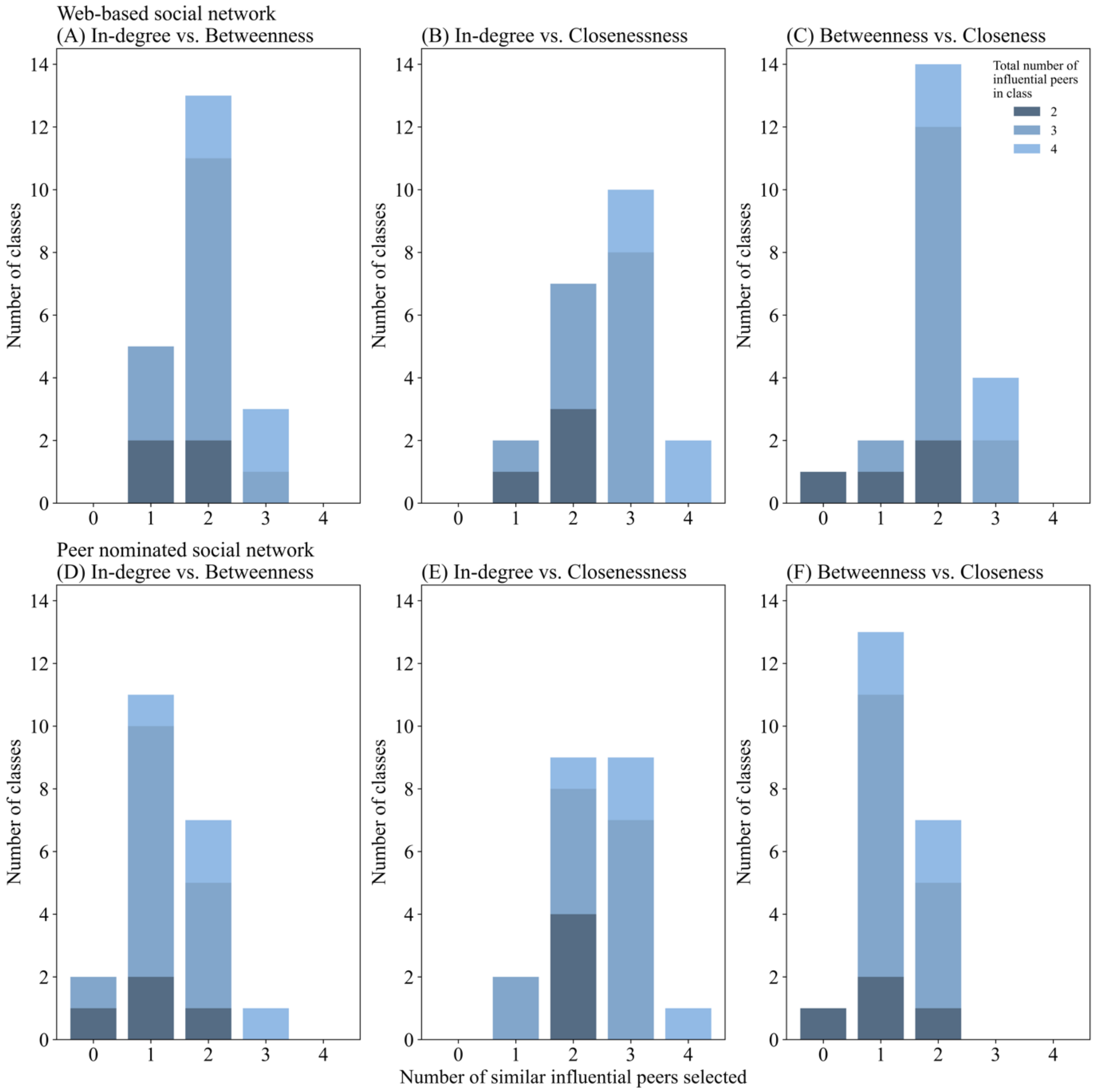

Note. Frequency distribution of similar influential peers for 1) in-degree vs. betweenness, 2) in-degree vs. closeness, and 3) betweenness vs. closeness. Panels A, B and C show results for web-based social networks, and panel D, E and F for peer nominated social networks. The overall similarity was 63% in panel A, 86% in panel B, 67% in panel C, 44% in panel D, 81% in panel E, 43% in panel F.
