# Supplementary material for: The Usefulness of Web-Based Communication Data for Social Network Health Interventions: Agent-Based Modeling Study
Source: JMIR Pediatr Parent. 2023 Nov 22;6:e44849. doi: 10.2196/44849 (PMC10701651; doi:10.2196/44849)
Supplement: Multimedia Appendix 8 [file pediatrics_v6i1e44849_app8.pdf]

Multimedia Appendix 8

Figure. Variation of the impact of social network interventions on physical activity levels between school classes.

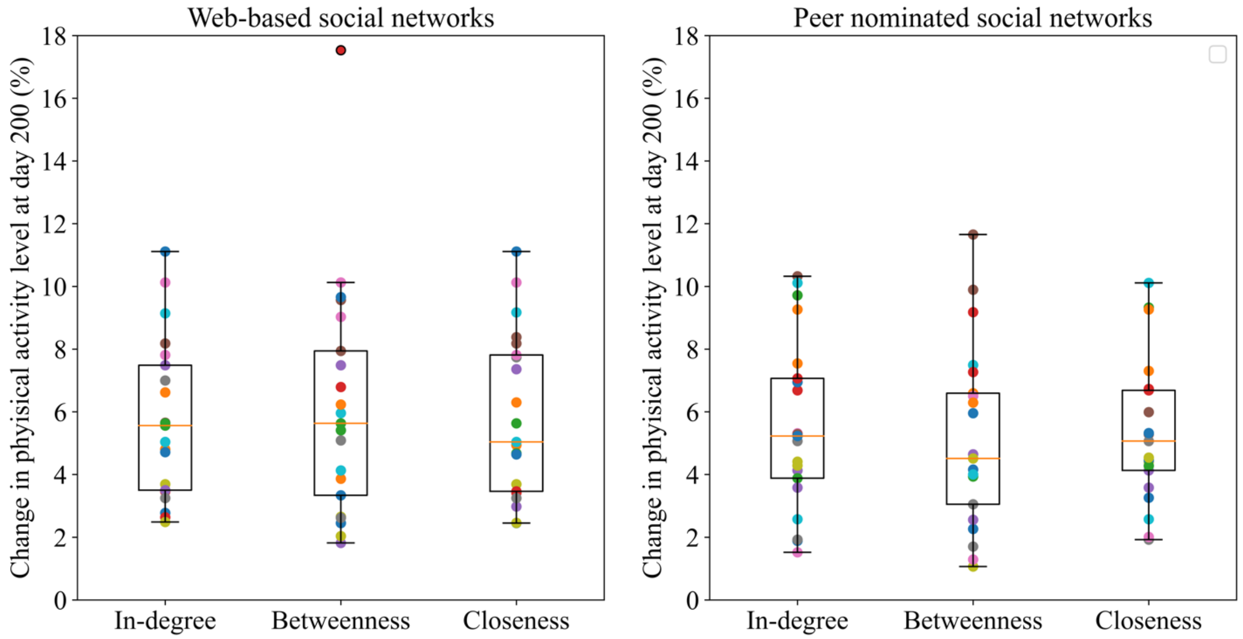

Note. The predicted mean effect of social network interventions on physical activity levels after 200 days. We assumed an increase of 17% in physical activity levels among influential peers who would spread the behavior. The colored points represent the mean change in physical activity level per class. Each color represents a school class. The points are overlaid with a boxplot to illustrate the variation in impact between school classes.

Table. The impact of network interventions on physical activity levels among adolescents per class after 200 days using social networks based on web-based communication and peer nomination data.

Class ID	Web-based social networks			Peer nominated social networks		
	In-degree %	Betweenness %	Closeness %	In-degree %	Betweenness %	Closeness %
1	2.8	2.5	2.5	1.9	2.3	3.3
2	4.8	3.9	4.9	7.5	6.6	7.3
3	5.6	5.4	4.7	9.7	3.9	9.3
4	2.6	17.5	3.4	6.7	9.2	6.7
5	7.5	7.5	7.4	4.1	4.6	4.1
6	5.7	9.6	8.4	10.3	11.7	6.0
7	10.1	10.1	10.1	5.3	6.5	5.3
8	7.0	5.1	7.7	1.9	1.7	1.9
9	3.7	2.7	3.7	4.4	1.1	4.4
10	9.1	6.0	9.2	10.1	7.5	10.1
11	4.7	3.3	4.6	6.9	4.2	4.5
12	6.6	6.2	6.3	9.3	6.3	9.3
13	5.6	5.6	5.6	3.9	3.9	4.3
14	3.5	6.8	3.5	7.1	7.3	6.7
15	3.5	1.8	3.0	3.6	2.6	3.6
16	8.2	7.9	8.2	5.3	9.9	5.3
17	7.8	9.0	7.8	1.5	1.3	2.0
18	3.2	2.6	3.2	5.1	3.1	5.1
19	2.5	2.0	2.5	4.3	4.5	4.5
20	5.0	4.1	5.0	2.6	4.0	2.6
21	11.1	9.7	11.1	5.2	6.0	5.3
