# Supplementary material for: The Usefulness of Web-Based Communication Data for Social Network Health Interventions: Agent-Based Modeling Study
Source: JMIR Pediatr Parent. 2023 Nov 22;6:e44849. doi: 10.2196/44849 (PMC10701651; doi:10.2196/44849)
Supplement: Multimedia Appendix 9 [file pediatrics_v6i1e44849_app9.pdf]

Multimedia Appendix 9

Figure. The impact of social network interventions using betweenness and closeness centrality on physical activity levels with varying effectiveness of health education for influential peers.

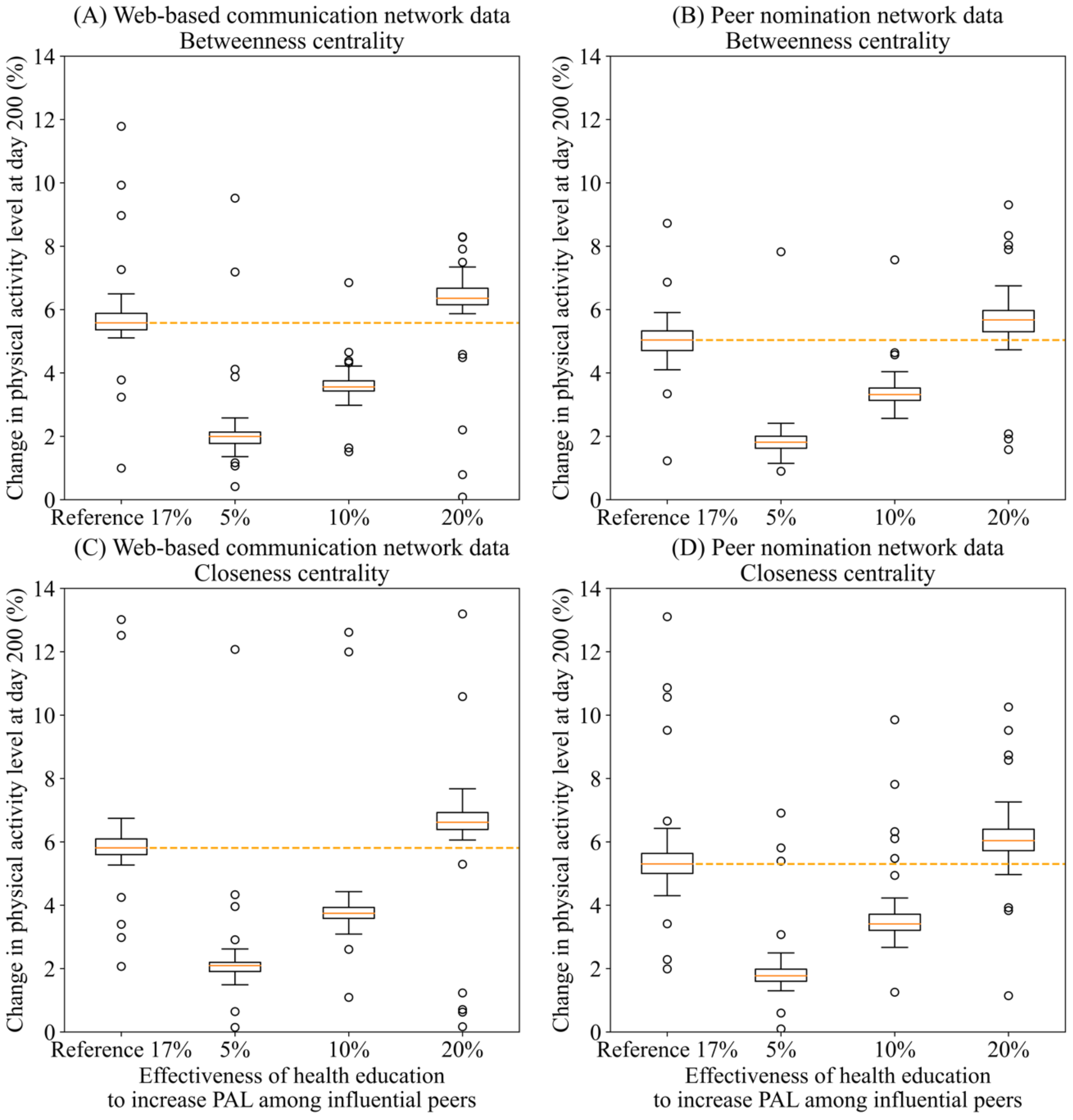

Note. Each panel shows the predicted impact of social network interventions on physical activity levels after 200 days based on web-based (A and C) and peer nominated social networks (B and D). We varied effectiveness of health education assuming an increase in physical activity levels among influential peers by 17% (reference), 5%, 10% and 20%, who would spread the behavior. Panels A and B illustrate the impact for selecting influential peers based on betweenness centrality, and panels C and D on closeness centrality.
